# Development of prognostic nomogram model to predict syncope recurrence in children with vasovagal syncope

**DOI:** 10.3389/fcvm.2023.1099115

**Published:** 2023-03-08

**Authors:** Rui Sun, Yingying Kang, Mingming Zhang, Hongmao Wang, Lin Shi, Xiaohui Li

**Affiliations:** ^1^ Department of Cardiology, Capital Institute of Pediatrics-Peking University Teaching Hospital, Beijing, China; ^2^ Department of Cardiology, Children’s Hospital Capital Institute of Pediatrics, Beijing, China; ^3^ Graduate School of Peking Union Medical College, Capital Institute of Pediatrics, Beijing, China

**Keywords:** vasovagal syncope, children, recurrence of syncope, prognosis, nomogram model

## Abstract

**Backgrounds:**

Vasovagal syncope (VVS) is a common form of syncope. In children with VVS, recurrent syncope or presyncope can affect the physical and mental health of both children and parents, which markedly impairs quality of life.

**Objectives:**

We aimed to identify factors at baseline that can predict the recurrence of syncope or presyncope over a 5-year follow-up period, and further to develop a prognostic nomogram model.

**Methods:**

This cohort is bidirectional in design. From July 2017 to August 2022, children with VVS were included and followed up every 3 to 6 months. Head-up Tilt Test (HUTT) was performed for diagnosing VVS. Data were analyzed using STATA software, and risk estimates are presented as hazard ratio (HR) and 95% confidence interval (CI).

**Results:**

Total 352 children with VVS who had complete information were included in this study. Median follow-up time was 22 months. Overall, supine mean arterial pressure (MAP-supine) in HUTT and baseline urine specific gravity (USG) were associated with the significant risk of syncope or presyncope recurrence (HR: 0.70 and 3.00, respectively; both *P* < 0.05). Calibration and discrimination analyses revealed that the addition of MAP-supine and USG can result in a better fit. A prognostic nomogram model based on significant factors annexed with five traditional promising factors was finally constructed, with strong discriminative and predictive abilities (C-index approaching 0.700, *P* < 0.05).

**Conclusions:**

Our findings indicated that MAP-supine and USG can independently predict the significant risk of syncope recurrence in children with VVS, and the prediction was more obvious in a nomogram model.

## 1. Introduction

Syncope is a major public health concern with approximately 17% of children 2 to 18 years of age experiencing at least one episode of syncope (1). As a common form of syncope, vasovagal syncope (VVS) accounts for about 60 to 80% of all pediatric cases (2). VVS tends to have a good prognosis, yet its recurrent episodes can affect the physical and mental health of children, as well as their parents, which markedly impairs the quality of life (3). As the development of syncope is a complex process, it is of clinical and healthcare importance to identify potential factors attributable to the recurrence of syncope in children affected with VVS.

Currently, research on the risk profiles of recurrent syncope or presyncope in children with VVS has been less conducted in medical literature. For example, some investigators have reported that the number of previous episodes of syncope (4, 5), onset age of syncope, and family history (6) were identified as promising predictors for recurrent syncope in VVS. In addition, others revealed that venous blood routine hemoglobin concentration and mean corpuscular hemoglobin were candidate biomarkers in predisposition to syncope recurrence (7). Further, heart rate variability (HRV), body mass index (BMI), and therapeutic regimens can also affect the prognosis of VVS (8–10). Thus far, no consensus has been attained which and how many factors actually precipitate the development of recurrent syncope in children with VVS, likely due to differences in genetic underpinnings, statistical power or participant characteristics. Given that VVS etiology is complex and usually involve a multistep, multifactorial process, the impact of any single factors is believed to be small, but may be more evident when assessed in combination. However, a literature search did not trace any hints concerning the joint contribution of various factors to recurrent syncope in VVS.

To fill this gap in knowledge and yield more references for future studies, we, in a large cohort of children with VVS, aimed to identify factors at baseline that can predict the recurrence of syncope over a 5-year follow-up period, and meanwhile for clinical application, to develop a prognostic nomogram model based on promising significant attributes.

## 2. Methods

### 2.1. Ethical approval and informed consent

The conduct of this study was approved by the Ethics Committee of Children's Hospital Capital Institute of Pediatrics (SHERLLM2021025), and written informed consent was obtained from parents or supervisors of participating children.

### 2.2. Study children

The present cohort is bidirectional in design. From July 2017 to August 2022, children diagnosed with VVS were hospitalized for the first time at the Department of Cardiology, Children's Hospital Capital Institute of Pediatrics, and after discharge they were followed up at the Out-Patient Department every 3–6 months. The latest follow-up was competed on October 7, 2022. In case of no-show at the scheduled time, we reached out to their parents or supervisors through phone. The minimal follow-up time was set at 1 month.

### 2.3. Inclusion and exclusion criteria

Children were included if all of the following criteria were satisfied simultaneously: (i) <18 years of age; (ii) hospitalized children; (iii) confirmed diagnosis of VVS. The clinical diagnostic criteria of VVS refer to the expert consensus on the diagnosis of syncope in children (11).

Children were excluded if they complicated any of the following criteria: (i) cardiovascular diseases, such as hypertension, arrhythmias, and congenital heart disease; (ii) neuropsychological diseases, such as anxiety and depression; (iii) metabolic diseases, such as hyperthyroidism and metabolic syndrome; (iv) convulsant syncope confirmed as seizure based on their medical histories as well as electroencephalograph and brain magnetic resonance imaging examination.

### 2.4. Follow-up assessment

The details of treatment regimens for children with VVS can be found in the Supplementary Materials (Appendix A.1). During each follow-up, autonomic exercises, oral rehydration salts, and drug regimens were recorded. Disease-specific survival was calculated from the date of diagnosis to the date of syncope or presyncope recurrence or the date of the last follow-up visit, whichever came first. Presyncope is defined as symptoms before syncope, and it includes extreme lightheadedness, visual sensations (such as tunnel vision and graying out), and variable degrees of altered consciousness without complete loss of consciousness. If the child had a transient loss of consciousness associated with inability to maintain postural tone with rapid and spontaneous recovery, syncope was confirmed. Recurrence information was obtained from either parents or supervisors. Children with VVS were divided into two groups: children with syncope or presyncope recurrence and children free of syncope or presyncope recurrence.

### 2.5. Head-up tilt test

Participating children were requested to lie on the electric tilting bed (SHUT -100A, STANDARD, Beijing, China) in the supine position for 10 min. A baseline head-up tilt test (HUTT) was conducted with the child tilted for 45 min without medication. If syncope did not develop, the child was given a sublingual nitroglycerin (4–6 *μ*g/kg, maximum ≤0.3 mg) to provoke syncope within 20 min. Automatic cuff blood pressure, heart rate, and electrocardiograph were recorded continuously by electric tilting instrument. If syncope occurred during the test, the tilt table would be quickly lowered to the supine position. Positive responses to HUTT involve syncope or presyncope in addition to four key factors, including (i) drops in blood pressure (systolic blood pressure [SBP] ≤80 mmHg diastolic blood pressure [DBP] ≤50 mmHg; (ii) a sinus arrest with junctional escape rhythm; (iii) an atrioventricular (AV) conduction block no less than II° or a cardiac arrest for three seconds; (iv) a decrease in heart rate of <75 beats per minute(bpm) in children aged 4–6 years, <65 bpm in those aged 7–8 years, and <60 bpm in those aged 8 years (11–13). In addition, VVS is divided into three types according to the changes in blood pressure and heart rate. The cases with lower blood pressure were defined as the vaso inhibitive type, the cases with significantly lower heart rate were defined as the VVS cardiac inhibitory type, and the cases with both lower heart rate and lower blood pressure as the VVS mixed type.

### 2.6. Data collection

For children admitted for the first time to our hospital, a standardized questionnaire was adopted to glean demographic information at baseline, including age, sex, BMI, medical history, number of syncope, and family history of syncope. Routine blood tests, cardiac enzymes, urine routine, 24-hour urine output and electrolytes, cardiac function, and 24-hour Holter during hospitalization were also recorded. All above tests were completed prior to the HUTT test. The first morning urine specimens were measured using an automatic urinary sediment analyzer (AX-4030, Arkray, Kyoto, Japan). Heart rate and arterial pressure were measured twice after affected children had stayed in a supine position for 10 min and again at 60-degree tilting when stable within the first 1 min.

In addition, the following indicators were also collected. Mean arterial pressure in the supine position for 10 min (MAP-supine), and mean arterial pressure in the first 1 min at 60 degrees of inclination (MAP-tilt) were calculated SBP and DBP in tilt position. MAP was calculated as DBP plus 0.333 × (SBP - DBP). Change-SBP was calculated as SBP in tilt immediately minus SBP in supine, and Change-DBP as DBP in tilt immediately minus DBP in supine. Change-HR was calculated as heart rate in tilt immediately minus heart rate in supine.

### 2.7. Statistical analysis

Continuous parameters are presented as mean ± standard deviation (SD) or as median (interquartile range) depending on whether the parameter was deviated from the Gaussian distribution. Where appropriate, the *t*-test or Mann-Whitney test was used to compare differences of continuous parameters between groups. Categorical parameters are presented as percentage (count) and compared using the *χ*^2^ test or Fisher's exact test. Potential factors related to recurrence were initially identified by the univariate Cox proportional hazards regression analyses. Then, the multivariate Cox proportional hazards regression analyses were undertaken to control for covariates, including age, sex, BMI, medical history, number of syncope, family history of syncope, and therapeutic regimens.

A series of discrimination and calibration statistics were used to evaluate prediction accuracy. To justify the improvement in prediction accuracy, net reclassification improvement (NRI) and integrated discrimination improvement (IDI) (14, 15) were calculated. If the outcome is less than 0.05, adding significant risk factors might greatly increase the model's ability in prediction. The area under the Harrell C-statistic is to examine whether the traditional model can distinguish between children who had presyncope or syncope recurrence when significant factors are added. To determine how closely the prediction probability for the addition of significant factors reflected actual observed risk and the overall fit of the improved risk model, the Akaike information criterion (AIC), Bayesian information criterion (BIC), and likelihood ratio test were utilized (16). For the AIC and BIC indices, smaller values indicate better models.

By using decision curve analysis (DCA), the net benefit of the addition of significant factors over the conventional model was clearly demonstrated (17). The DCA graph's X-axis represents the recurrence thresholds, while the Y-axis represents the net benefits for various thresholds. If the “model” curve is further from the solid curve line under all recurrence assumptions and the dotted horizontal line with none recurrence assumptions, a bigger net benefit is reported.

A predicted nomogram for the 1-year, 2-year, and 3-year recurrence rates from VVS was constructed among all research patients based on baseline demographic and clinical data with statistical significance, improving clinical interpretation. The predictive precision and discriminative power of this prognostic nomogram were assessed using the concordance index (C-index) and calibration curve. The area under the receiver operating characteristic curve and the C-index are equivalent. According to the calibration curve, the 45° line illustrates how far the prognostic nomogram's predicted probabilities differ from actual outcomes.

The regression modeling strategies (RMS) package (https://cran.r-project.org/web/packages/rms/index.html) in the R- language (version 4.2.1) was used to create the prognostic nomogram model. A nomogram is a graphic calculator that has been scaled and is used to determine an approximation of a function. In this study, a simple 10-point scale was used as the basis for the nomogram.

All of the aforementioned statistical analyses were conducted using STATA/SE software (version 14.0, Stata Corp, TX, USA). Probability less than 5% was considered statistically significant.

## 3. Results

### 3.1. Sample size

In total, 409 children were diagnosed with VVS during the study period, and 37 of them were excluded because of anxiety and depression (*n* = 23), hyperthyroidism (*n* = 7), arrhythmias (*n* = 3), hypertension (*n* = 3), and abnormal origin coronary artery (*n* = 1). Twenty children were lost during follow-up, leaving 352 patients with complete follow-up data in the final analysis. The median time to an endpoint (syncope or presyncope recurrence) event occurred was 22 months (from 1 day to 63 months).

### 3.2. Baseline characteristics

The baseline characteristics of study patients are showed in Table 1. Urine specific gravity (USG) before diagnosis and the time used oral rehydration salts (ORS) during follow-ups differed significantly between children with or without recurrence (*P* < 0.05). No significance was noted for the rest characteristics.

**Table 1:** Baseline characteristics of participating children by the presence and absence of recurrence of vasovagal syncope.

	No Recurrence (*n* = 220)	Recurrence (*n* **= **132)	*P*
**Demographic information**			
Age (years)	12.00 (9.29, 13.34)	12.00 (9.90, 13.64)	0.068
Girls, *n* (%)	120 (54.5)	84 (63.6)	0.118
Body mass index (kg/m^2^)	18.00 (15.76, 20.05)	18.00 (15.67, 19.89)	0.786
Medical history (months)	3.50 (0.75, 12.00)	6.00 (1.00, 24.00)	0.068
Number of syncope (*n*)	1.00 (0.00, 2.00)	1.00 (0.00, 2.00)	0.553
Family history of syncope (%)	22 (10.0)	18 (13.6)	0.303
**Laboratory Examination**			
Hemoglobin (g/L)	130.00 (124.00, 138.00)	130 (126.00, 137.25)	0.621
MCV (fL)	84.00 (81.85, 86.70)	84.00 (81.90, 87.32)	0.637
MCH (pg)	29.00 (27.80, 29.70)	29.00 (27.70, 29.80)	0.516
MCHC (g/L)	340.00 (332.50, 346.00)	340.00 (332.75, 346.00)	0.912
Creatine Kinase (U/L)	74.00 (60.00, 98.00)	70.00 (54.50, 91.50)	0.119
Creatine Kinase-MB (ng/ml)	0.54 (0.21, 0.90)	0.45 (0.20, 0.80)	0.135
Urine output (ml/24 h)	1300.00 (894.00, 1,755.00)	1300.00 (950.00, 1,748.00)	0.888
Total-Na (mmol/24 h)	120.00 (93.06, 170.13)	120.00 (78.57, 171.70)	0.728
Total-K (mmol/24 h)	31.00 (24.86, 39.90)	30.00 (22.36, 37.59)	0.309
Urine specific gravity	1.02 (1.01, 1.02)	1.02 (1.01, 1.03)	0.016
LVEF (%)	70.00 (67.00, 73.50)	70.00 (66.00, 73.00)	0.674
LVFS (%)	39.00 (36.00, 42.00)	39.00 (36.00, 42.00)	0.662
**24-Hour Holter**			
Average HR (bpm)	81.00 (75.00, 86.00)	80.00 (75.00, 87.00)	0.733
SDNN (ms)	150.00 (127.00, 175.00)	150.00 (132.00, 172.00)	0.878
SDANN (ms)	130.00 (110.00, 151.00)	120.00 (111.00, 148.00)	0.819
SDNN index (ms)	73.00 (60.00, 87.25)	72.00 (61.00, 88.00)	0.961
pnn50 (%)	24.00 (15.00, 33.00)	22.00 (16.00, 32.00)	0.493
DC	7.10 (6.27, 8.01)	7.20 (6.46, 7.95)	0.687
TP	4200.00 (3,049.80, 5,895.57)	3900.00 (2,994.78, 5,596.25)	0.172
LF/HF	1.50 (1.17, 2.06)	1.60 (1.24, 2.00)	0.367
**Head-up Tilt Test**			
MAP-supine (mm Hg)	81.00 (75.58, 86.00)	79.00 (75.33, 84.00)	0.188
MAP-tilt (mm Hg)	80.00 (74.67, 85.00)	78.00 (73.00, 83.00)	0.154
Change-SBP (mm Hg)	−1 (−7.00, 3.00)	0 (−6.00, 4.00)	0.398
Change-DBP (mm Hg)	−3 (−8.00, 1.00)	−5 (−9.00, 1.00)	0.06
Change-HR (bpm)	17.00 (11.00, 24.00)	16.00 (10.00, 24.25)	0.995
Positive reaction time (min)	35.00 (20.00, 35.00)	33.00 (18.00, 35.00)	0.332
Nitroglycerin (%)	140 (63.6)	78 (59.5)	0.515
**Type (%)**			
Type1	86 (39.1)	53 (40.2)	0.262
Type2	46 (20.9)	36 (27.3)	
Type3	88 (40.0)	43 (32.6)	
Sinus arrest (%)	9 (4.1)	9 (6.8)	0.319
**Therapy**			
Autonomic nerve function exercises (%)	144 (65.5)	76 (57.6)	0.142
ORS (months)	1.00 (0.25, 2.00)	1.00 (0.00, 3.50)	0.029
**Drug therapy (%)**			
Without	158 (71.8)	88 (66.7)	0.578
Metoprolol	46 (20.9)	32 (24.2)	
Sertraline hydrochloride	16 (7.3)	12 (9.1)	
Drug therapy duration (months)	0 (0.00, 1.00)	0 (0.00, 1.00)	0.292

SBP, systolic blood pressure; DBP, diastolic blood pressure; MAP, average arterial pressure; HR, heart rate; MCV, mean corpuscular volume; MCH, mean corpuscular hemoglobin; MCHC, mean corpuscular hemoglobin concentration; LVEF, left ventricular ejection fraction; LVFS, left ventricular fraction shortening; SDNN, standard deviation of RR intervals in milliseconds; SDANN, Standard deviation of the average RR intervals milliseconds; SDNN index, Mean score of the standard deviations of all RR intervals in 5-min segments in milliseconds; pnn50, Proportion of pairs of successive RR intervals differing by more than 50 ms divided by the total number of RR intervals (percentage); DC, deceleration capacity; TP, total power, the frequency components in heart rate variability; LF/HF, ratio between the low- and high frequency component; Type1, vasodepressor type; Type2, cardioinhibitory type; Type3, mixed type; ORS, oral rehydration salts. Sinus arrest means the children had syncope with sinus arrest during an inspection in the hospital. “Autonomic nerve function exercises” doesn’t involve the use of MSNA.

Comparison of baseline characteristics between children analyzed and lost to follow-ups is provided in Supplementary Table S1.

### 3.3. Identification of significant factors

The two factors, MAP-supine and USG, were associated with the significant risk of syncope or presyncope recurrence in VVS patients before adjustment at a 5% level of significance (Table 2). After adjusting for age, sex, BMI, medical history, number of syncope, family history of syncope, and therapeutic regimens, three factors were consistently and independently associated with the significant risk of recurrence in VVS, including MAP-supine (HR, 95% CI, *P*: 0.700, 0.567 to 0.869, 0.001), MAP-tilt (0.796, 0.654 to 0.969, 0.035), and USG (3.000, 1.03 to 8.741, 0.044).

**Table 2:** Identification of factors independently and significantly associated with the recurrence of vasovagal syncope.

Factors at baseline	Univariate model			Multivariable model		
	HR	95% CI	*P*	HR	95% CI	*P*
Age	1.005	0.999 to 1.011	0.090	NA	NA	NA
Sex	0.738	0.517 to 1.052	0.093	NA	NA	NA
BMI	0.993	0.943 to 1.047	0.801	NA	NA	NA
Medical history	1.064	0.986 to 1.149	0.111	NA	NA	NA
Number of syncope	1.013	0.926 to 1.109	0.778	NA	NA	NA
Family history of syncope	1.120	0.68 to 1.844	0.656	NA	NA	NA
Hemoglobin	1.001	0.987 to 1.016	0.899	1.004	0.988 to 1.021	0.601
MCV	1.000	0.997 to 1.002	0.750	1.000	0.996 to 1.003	0.769
MCH	1.042	0.953 to 1.14	0.365	1.026	0.937 to 1.123	0.580
MCHC	0.991	0.976 to 1.006	0.226	0.991	0.976 to 1.007	0.285
Creatine Kinase	0.995	0.989 to 1.000	0.067	0.996	0.99 to 1.002	0.250
Creatine Kinase-MB	0.908	0.7 to 1.178	0.469	0.951	0.756 to 1.195	0.665
LVEF	0.999	0.958 to 1.042	0.969	1.008	0.966 to 1.052	0.705
LVFS	0.997	0.948 to 1.049	0.912	1.007	0.957 to 1.06	0.778
Urine output of 24h	1.000	1.0 to 1.0	0.889	1.0	1.0 to 1.0	0.901
Total-Na	1.000	0.998 to 1.002	0.857	1.000	0.998 to 1.002	0.990
Total-K	1.000	1.0 to 1.001	0.298	1.000	1.0 to 1.001	0.258
**Urine specific gravity (+0.05)**	3.455	1.232 to 9.691	0.018	3.000	1.03 to 8.741	0.044
Average heart rate	0.999	0.982 to 1.018	0.952	1.001	0.979 to 1.024	0.912
SDNN	0.999	0.996 to 1.003	0.638	1.000	0.996 to 1.003	0.957
SDANN	1.000	0.994 to 1.005	0.943	1.000	0.994 to 1.006	0.919
SDNN index	0.998	0.992 to 1.003	0.421	0.999	0.993 to 1.005	0.710
pnn50	0.997	0.983 to 1.011	0.684	1.000	0.987 to 1.014	0.988
DC	0.998	0.977 to 1.02	0.863	0.994	0.882 to 1.121	0.925
TP	1.000	1.0 to 1.0	0.343	1.000	1.0 to 1.0	0.154
LF/HF	1.093	0.874 to 1.367	0.435	1.123	0.885 to 1.424	0.339
**MAP-supine** **(+10 mmHg)**	0.775	0.633 to 0.948	0.013	0.700	0.567 to 0.869	0.001
**MAP-tilt** (**+10 mmHg**)	0.879	0.734 to 1.053	0.161	0.796	0.654 to 0.969	0.035
Change-SBP (+10 mmHg)	1.050	0.879 to 1.254	0.589	1.096	0.911 to 1.319	0.332
Change-DBP (+10 mmHg)	0.876	0.714 to 1.076	0.207	0.863	0.71 to 1.048	0.138
Change-HR (+10 bpm)	0.863	0.71 to 1.048	0.138	0.876	0.714 to 1.076	0.207
Positive reaction time (min)	0.991	0.976 to 1.007	0.272	0.993	0.977 to 1.009	0.407
Nitroglycerin	0.816	0.592 to 1.125	0.214	0.800	0.578 to 1.109	0.181
Type	0.820	0.671 to 1.004	0.054	0.820	0.663 to 1.015	0.068
Sinus arrest	1.354	0.688 to 2.666	0.380	0.778	0.296 to 2.046	0.611

HR, hazard ratio; 95% CI, 95% confidence interval; SBP, systolic blood pressure; DBP, diastolic blood pressure; MAP, average arterial pressure; MCV, mean corpuscular volume; MCH, mean corpuscular hemoglobin; MCHC, mean corpuscular hemoglobin concentration; LVEF, left ventricular ejection fraction; LVFS, left ventricular fraction shortening; SDNN, standard deviation of RR intervals in milliseconds; SDANN, Standard deviation of the average RR intervals milliseconds; SDNN index, Mean score of the standard deviations of all RR intervals in 5-min segments in milliseconds; pnn50, Proportion of pairs of successive RR intervals differing by more than 50 ms divided by the total number of RR intervals (percentage); DC, deceleration capacity; TP, total power, the frequency components in heart rate variability; LF/HF, ratio between the low- and high frequency component; ORS, oral rehydration salts, NA, not available. Sinus arrest means that children had syncope with sinus arrest during an inspection in the hospital. In multivariable model, adjusted factors included age, sex, body mass index, medical history, number of syncope, family history of syncope, and therapeutic regimens.

### 3.4. Prediction accuracy assessment

Because MAP-supine had collinearity with MAP- tilt, MAP- tilt was removed from the following analyses. To evaluate the predictive power of the major factors identified, two models—the basic model and the full model—were built. The basic model includes general characteristics (age, sex, BMI, medical history, family history of syncope), and the full model includes general characteristics and the two significant factors (MAP-supine and USG).

The prediction accuracy gained by adding the two major factors to the basic model was assessed using both calibration and discrimination statistics (Table 3). Compared with the basic model, the prediction accuracy of the full model was significantly improved. The AIC and BIC in full model were smaller, and as revealed by the likelihood ratio test, both models differed significantly in prediction performance (*P* = 0.001). NRI and IDI statistics showed significant improvement after adding MAP-supine and USG to the basic model from the aspect of reclassification performance. Harrell's C-statistic indicated that the addition of MAP-supine and USG can improve the predictive ability of the basic model.

**Table 3:** Predictive accuracy of risk model with and without MAP-supine or USG for the prediction of VVS recurrence.

Statistics	Basic model	Full model
***Calibration***		
AIC	1,432.398	1,217.030
BIC	1,455.580	1,246.897
LR test (*χ*^2^)	13.670	
LR test (*P* value)	0.001	
***Discrimination***		
NRI (*P* value)	0.043	
IDI (*P* value)	0.000	
Harrell's C	0.589	0.651

VVS, vasovagal syncope; MAP, average arterial pressure; USG, urine specific gravity; AIC, Akaike information criterion; BIC, Bayesian information criteria; LR test, likelihood ratio test; NRI, net reclassification improvement; IDI, integrated discrimination improvement. Basic model included age, sex, body mass index, medical history, number of syncope, and family history, and full model additionally included MAP-supine and USG.

**Figure 1:**
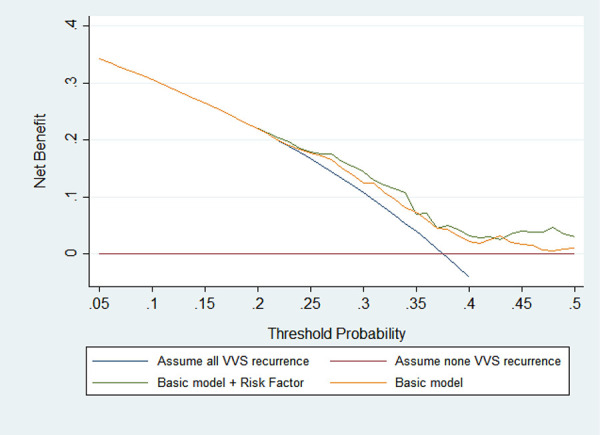
Prognostic decision curves for the basic model and the addition of MAP-supine and urine specific gravity in vasovagal syncope patients. The basic model included age, sex, body mass index, medical history, and family history.

Furthermore, DCA graph indicated the obvious net benefits gained by adding the two significant factors to the basic model (Figure 1).

### 3.5. Risk prognostic nomogram model

**Figure 2:**
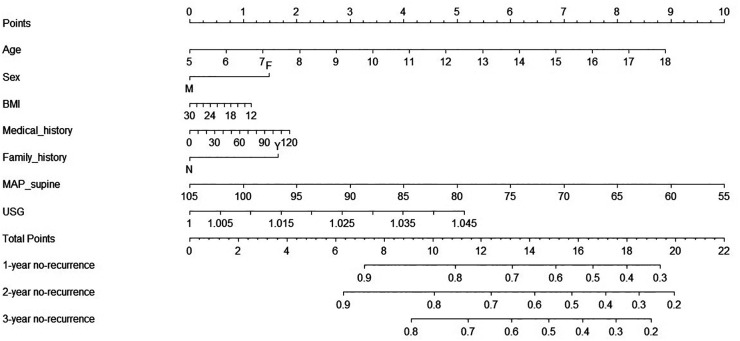
Prognostic nomogram of the prediction of significant parameters for 1-year, 2-year, and 3-year no-recurrence of vasovagal syncope among all study children. Abbreviations: F, female; M, male; Y, with family history of syncope; N, without family history of syncope; BMI, body mass index; MAP, average arterial pressure; USG, urine specific gravity.

To facilitate clinical interpretation, a prognostic nomogram model was constructed on the basis of the two significant factors aforementioned and some acknowledged conventional factors (Figure 2). For an example of clinical usefulness of this nomogram, assuming a girl (1.5 points) aged ten years (3.5 points), with a family history of syncope (1.8 points), BMI of 16 kg/m^2^ (1 points), MAP-supine 90 mmHg (2.9 points), and USG 1.025 (2.8 points), she would have an estimated 71% chance to experience no-recurrence of syncope or presyncope at 1st year and 65% chance at 2nd year, and 58% chance at 3rd year. Thus, the recurrence rate in 1st year, 2nd year, and 3rd year were estimated to be 29%, 35%, and 42%, respectively.

**Figure 3:**
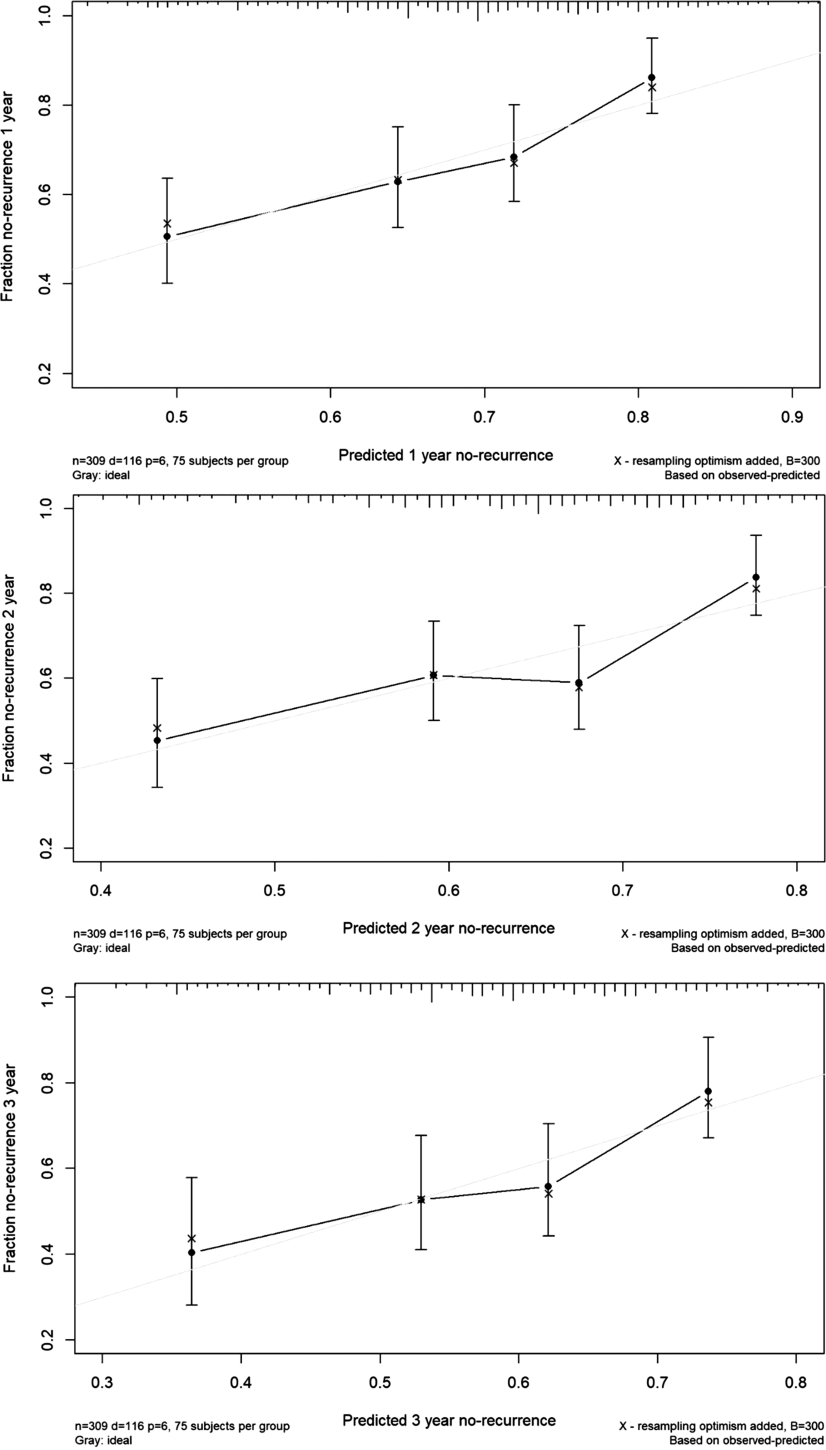
The calibration curves for predicting the risk of no-recurrence of vasovagal syncope at 1-year (the upper panel), 2-year (the middle panel) and 3-year (the lower panel) among all study children. Nomogram-predicted probability of no-recurrence is plotted on the X-axis, and actual no-recurrence is plotted on the Y-axis.

This nomogram had a good prediction accuracy, with the C-index approaching 70%. In addition, the calibration curves for the probabilities of no-recurrence at 1-year, 2-year, and 3-year diagnosed showed the best possible agreement between this prognostic nomogram's forecast and the actual observations (Figure 3).

## 4. Discussion

The aim of this cohort study was to identify factors at baseline that can predict the recurrence of syncope or presyncope in children with VVS and develop a prognostic nomogram model based on promising significant attributes. It is worth noting that MAP-supine and USG can independently predict the significant risk of syncope or presyncope recurrence in VVS, especially in a nomogram model annexed with 5 other established factors. As far as we know, this is thus far the largest study that has examined factors in a joint manner associated with syncope or presyncope recurrence in children with VVS in the literature.

It is widely recognized that age is a promising factor for syncope recurrence among adults with VVS, and the recurrence rates increase with aging (18, 19). Similarly in this study, our findings supported the contribution of age to syncope recurrence in children with VVS. A possible explanation is that the epinephrine (Epi)/norepinephrine (NE) ratio increases to a greater extent in younger fainters (20). Other factors, such as number of previous syncope and family history, also played a contributory role in the recurrence of syncope or presyncope in VVS. For example, in a Canadian study of 51 children with VVS the higher number of previous syncope was linked to the greater likelihood of syncope or presyncope recurrence (4). Another study from Turkey showed that age, family history, and several prior syncope onsets were factors attributable to syncope recurrence (6). Yet, in the present study, we failed to detect statistical significance for above demographic factors, and instead we focused on laboratory indicators by taking these factors as confounders. After a careful analysis among 352 children with VVS, our findings indicated that MAP-supine and USG were independently and significantly associated with the risk of syncope or presyncope recurrence in children with VVS, differing from the results of a previous study (21) of 63 children with VVS, which identified hemoglobin and MCH as two recurrence-predisposition factors. Given the complex nature of syncope recurrence, we agree that identification of more promising factors that can accurately predict VVS prognosis is still in the process of exploration, perfection and renewal.

In fact, USG, a non-invasive biomarker and objective substitutability for water homeostasis, are widely used in clinical practice (22). The USG fluctuates depending on the amount of fluids consumed, and it reflects the total amount of water consumed and lost (23, 24). An increase in USG indicates low water intake or excessive water loss. Prior studies have shown that USG had a specific predictive value in diagnosing VVS in children and adolescents (25). However, there is a paucity of data on its role in predicting syncope of presyncope recurrence. Our study showed that elevated USG was a statistically significant risk factor for syncope or presyncope recurrence in VVS, and for practical reasons, USG can be routinely monitored for the early identification of possible recurrence. Moreover, MAP is the average of arterial blood pressure during a cardiac cycle. The median or mean MAP was regularly higher than MAP of 65 mmHg (range 70–114 mmHg) (26, 27). Considering the fact that children are in a period of growth and development, blood pressure is determined based on age, gender, BMI, and so there is currently no uniform value for MAP. In the present study, lower the MAP-supine and MAP-tilt, were found to be associated with the greater likelihood of syncope or presyncope recurrence in VVS. Previous studies showed that children with VVS usually had high levels of catecholamine, which can cause excessive contraction of the heart and abnormal Bezold-Jarish reflex (10). Then, the imbalance of sympathetic impulses and vagal impulses may reduce MAP, which is commonly seen in our recurrence group. Lower MAP in the recurrence group in this study indicated that BP can be easily decreased in the presence of triggering factors, causing the symptoms of pre-syncope. Further decrease in BP annexed with sudden falls in cerebral blood flow can lead to syncope eventually (28, 29). Both USG and MAP were for the first time identified as predisposing factors for syncope or presyncope recurrence in children with VVS, and more large-scale, well-designed studies are warranted to confirm or refute our findings.

Besides the obvious advantages, including large sample size, extended follow-up, and effective calibration/discrimination assessment, some possible limitations should be acknowledged. Firstly, because this study was conducted at a single site, our conclusions would be broadly applied pending consistently validated in other independent cohorts. Secondly, all assessable children with VVS were enrolled between July 2017 and August 2022, and during the 5-year period remarkable advances in techniques and deepened knowledge about this disease can introduce a potential bias that understates the influence of factors on the recurrence of syncope or presyncope. Thirdly, the findings presented here were based merely on children with VVS free of comorbidities and thereby cannot be extrapolated to all VVS populations. Last but not the least, only baseline biomarkers were assayed in this cohort, and frequently monitoring of these biomarkers and analyzing their dynamic variations may be of added interest. We agree that more investigations are warranted to confirm or refute the factors associated with VVS recurrence in this study.

## Conclusions

Taken together, our findings indicated that MAP-supine and USG can independently predict the significant risk of syncope or presyncope recurrence in VVS, and the prediction was more obvious in a nomogram model. For practical reasons, we hope that this study will not only serve as an endpoint but also a new beginning for future large, well-designed studies to explore the risk profiles of syncope or presyncope recurrence in children with VVS.

## Data Availability

The original contributions presented in the study are included in the article/**Supplementary Material**, further inquiries can be directed to the corresponding author/s.
