# Supplementary material for: Development of prognostic nomogram model to predict syncope recurrence in children with vasovagal syncope
Source: Front Cardiovasc Med. 2023 Mar 8;10:1099115. doi: 10.3389/fcvm.2023.1099115 (PMC10031040; doi:10.3389/fcvm.2023.1099115)
Supplement: Supplementary file 1 [file Table1.docx]

Supplementary Materials

# Supplementary Tables

**Table S1.** Baseline characteristics of children assessable and lost to follow-up

| **Characteristics** | **Assessable (n=352)** | **Lost (n=20)** | **P** |
| --- | --- | --- | --- |
| **Demographic information** |  |  |  |
| Age (years) | 12.00 (9.59, 13.50) | 12.00 (10.87, 13.73) | 0.683 |
| Girls, n (%) | 204 (58.00) | 14 (70.00) | 0.355 |
| Body-mass index (kg/m^2^) | 17.78 (15.72, 20.03) | 19.00 (17.65, 19.83) | 0.148 |
| Medical history (months) | 5.00 (1.00, 20.00) | 10.00 (1.92, 24.00) | 0.312 |
| Number of syncope | 1.00 (0.00, 2.00) | 1.00 (0.00, 2.25) | 0.814 |
| Family history of syncope (%) | 40 (11.4) | 2 (10.0) | 1.000 |
| **Laboratory Examination** |  |  |  |
| Hemoglobin (g/L) | 130.00 (124.00, 138.00) | 130.00 (121.50, 135.50) | 0.572 |
| MCV (fL) | 84.00 (81.90, 87.00) | 84.00 (82.92, 86.78) | 0.565 |
| MCH (pg) | 29.00 (27.72, 29.70) | 29.00 (28.35, 29.45) | 0.485 |
| MCHC (g/L) | 340.00 (332.50, 346.00) | 340.00 (338.75, 345.00) | 0.335 |
| Creatine Kinase | 73.00 (58.25, 95.00) | 76.00 (67.50, 86.75) | 0.522 |
| Creatine Kinase-MB | 0.50 (0.20, 0.86) | 0.69 (0.19, 1.13) | 0.482 |
| Urine output (ml/24h) | 1300.00 (900.00, 1,754.75) | 1300.00 (938.00, 1,730.50) | 0.800 |
| Total-Na (mmol/24h) | 120.00 (85.57, 171.70) | 130.00 (92.72, 146.51) | 0.977 |
| Total-K (mmol/24h) | 30.00 (23.92, 39.30) | 37.00 (21.28, 40.41) | 0.693 |
| Urine specific gravity | 1.00 (1.01, 1.03) | 1.00 (1.01, 1.02) | 0.525 |
| LVEF (%) | 70.00 (67.00, 73.00) | 69.00 (66.75, 70.25) | 0.254 |
| LVFS (%) | 39.00 (36.00, 42.00) | 38.00 (36.00, 39.25) | 0.525 |
| **24-Hour Holter** |  |  |  |
| Average HR | 81 (75.00, 86.00) | 83 (79.00, 89.50) | 0.137 |
| SDNN (ms) | 150.00 (129.00, 175.00) | 140.00 (121.75, 170.75) | 0.351 |
| SDANN (ms) | 130.00 (110.00, 149.00) | 120.00 (99.00, 145.25) | 0.378 |
| SDNN index (ms) | 73.00 (60.00, 88.00) | 66.00 (60.00, 87.00) | 0.555 |
| pnn50 (%) | 23.00 (15.00, 32.00) | 22.00 (15.00, 31.00) | 0.782 |
| DC | 7.10 (6.30, 8.00) | 7.10 (6.07, 8.11) | 0.964 |
| TP | 4100.00 (3,015.68, 5,786.62) | 4100.00 (3,456.80, 5,413.12) | 0.770 |
| LF/HF | 1.50 (1.20, 2.04) | 1.30 (0.99, 1.84) | 0.148 |
| **Head-up Tilt Test** |  |  |  |
| MAP-supine (mmHg) | 80.00 (75.33, 85.50) | 81.00 (76.75, 83.58) | 0.845 |
| MAP-tilt (mmHg) | 79.00 (74.17, 84.33) | 80.00 (75.33, 84.08) | 0.814 |
| Change-SBP (mmHg) | -1.00 (-6.00, 3.50) | -0.50 (-6.25, 3.00) | 0.808 |
| Change-DBP (mmHg) | -4.00(-8.00, 1.00) | -4.00(-7.25, -1.50) | 0.124 |
| Change-HR (mmHg) | 17.00 (10.75, 24.00) | 16.00 (10.00, 25.25) | 0.985 |
| Positive reaction time (min) | 35.00 (19.50, 35.00) | 34.00 (23.75, 35.00) | 0.770 |
| Nitroglycerin (%) | 218 (62.1) | 13 (65.0) | 1.000 |
| Type (%) |  |  |  |
| Type1 | 139 (39.5) | 7 (35.0) | 0.515 |
| Type2 | 82 (23.3) | 7 (35.0) |  |
| Type3 | 131 (37.2) | 6 (30.0) |  |
| Sinus arrest (%) | 18 (5.1) | 0 (0.0) | 0.612 |

Abbreviations: SBP, systolic blood pressure; DBP, diastolic blood pressure; MAP, average arterial pressure; HR, heart rate; MCV, mean corpuscular volume; MCH, mean corpuscular hemoglobin; MCHC, mean corpuscular hemoglobin concentration; LVEF, left ventricular ejection fraction; LVFS, left ventricular fraction shortening; SDNN, standard deviation of RR intervals in milliseconds; SDANN, Standard deviation of the average RR intervals milliseconds; SDNN index, Mean score of the standard deviations of all RR intervals in 5-min segments in milliseconds; pnn50, Proportion of pairs of successive RR intervals differing by more than 50ms divided by the total number of RR intervals (percentage); DC, deceleration capacity; TP, total power, the frequency components in heart rate variability; LF/HF, ratio between the low- and high frequency component; Type1, vasodepressor type; Type2, cardioinhibitory type; Type3, mixed type; ORS, oral rehydration salts. Sinus arrest means the children had syncope with sinus arrest during an inspection in the hospital.

# Appendix

**A.1 Treatment for VVS patients**

According to the guideline recommended: Children with VVS had both non-pharmacological and pharmaceutical therapy options (1-3). Non-pharmacological treatment included education, exercise of autonomic nervous function, and increased intake of water and salt. Education refers to encourage the child to stay away from things that can cause syncope, like stuffy environments and prolonged standing, and to stay squatting or lying down in a secure location whenever symptoms like dizziness, vertigo, and amaurosis appear. Exercise of autonomic nervous function includes tilt training and wiping the limbs with a dry towel. For tilt training, children are required to stand against the wall at home, with their feet 15 cm away from the wall. Gradually increase the standing time, 1-2 times a day according to the child's tolerance (up to 30 minutes for each training). To avoid fainting or fainting-like injuries, a family member can walk with children during the tilt training. Oral rehydration salts (ORS; recommended 1 pack of 5.125 g/pack per day): the content of each pack can be dissolved in 250-500 ml of water to supplement water and salt intake.

Children with recurrent episodes (≥ 2 times within six months or ≥ 3 times within 1 year) and a risk of trauma can be considered for medical treatment (1-3). Metoprolol was administered to patients whose heart rates increased by more than 30 beats per minute prior to a positive response during HUTT. Sertraline hydrochloride was administered to patients in our study who had a history of repeated syncope episodes.

References

1. Brignole M, Moya A, de Lange FJ, Deharo J-C, Elliott PM, Fanciulli A, et al. 2018 ESC Guidelines for the diagnosis and management of syncope. European Heart Journal. 2018;39(21):1883-948.

2. Du J. Expert consensus on the treatment of vasovagal syncope and postural tachycardia syndrome in children. Chinese Journal of Pediatrics. 2018;56:6-9.

3. Shen W-K, Sheldon RS, Benditt DG, Cohen MI, Forman DE, Goldberger ZD, et al. 2017 ACC/AHA/HRS guideline for the evaluation and management of patients with syncope: a report of the American College of Cardiology/American Heart Association Task Force on Clinical Practice Guidelines and the Heart Rhythm Society. Journal of the American College of Cardiology. 2017;70(5):e39-e110.
